# Effect of Rearing Temperature on Growth and Microbiota Composition of *Hermetia illucens*

**DOI:** 10.3390/microorganisms8060902

**Published:** 2020-06-15

**Authors:** Stefano Raimondi, Gloria Spampinato, Laura Ioana Macavei, Linda Lugli, Francesco Candeliere, Maddalena Rossi, Lara Maistrello, Alberto Amaretti

**Affiliations:** 1 Department of Life Sciences, University of Modena and Reggio Emilia, 41125 Modena, Italy; stefano.raimondi@unimore.it (S.R.); gloria.spampinato@unimore.it (G.S.); lauraioana.macavei@unimore.it (L.I.M.); lugli.linda@gmail.com (L.L.); francesco.candeliere@unimore.it (F.C.); maddalena.rossi@unimore.it (M.R.); lara.maistrello@unimore.it (L.M.); 2 BIOGEST–SITEIA, University of Modena and Reggio Emilia, 42124 Reggio Emilia, Italy

**Keywords:** *Hermetia illucens*, microbiological risk assessment, black soldier fly, microbiota, 16S rRNA gene, metagenome

## Abstract

The potential utilization of black soldier fly (*Hermetia illucens*) as food or feed is interesting due to the nutritive value and the sustainability of the rearing process. In the present study, larvae and prepupae of *H. illucens* were reared at 20, 27, and 33 °C, to determine whether temperature affects the whole insect microbiota, described using microbiological risk assessment techniques and 16S rRNA gene survey. The larvae efficiently grew across the tested temperatures. Higher temperatures promoted faster larval development and greater final biomass but also higher mortality. Viable Enterobacteriaceae, *Bacillus cereus*, *Campylobacter*, *Clostridium perfringens*, coagulase-positive staphylococci, *Listeriaceae*, and *Salmonella* were detected in prepupae. *Campylobacter* and *Listeriaceae* counts got higher with the increasing temperature. Based on 16S rRNA gene analysis, the microbiota of larvae was dominated by *Providencia* (>60%) and other *Proteobateria* (mainly *Klebsiella*) and evolved to a more complex composition in prepupae, with a bloom of *Actinobacteria*, *Bacteroidetes*, and *Bacilli*, while *Providencia* was still present as the main component. Prepupae largely shared the microbiota with the frass where it was reared, except for few lowly represented taxa. The rearing temperature was negatively associated with the amount of *Providencia*, and positively associated with a variety of other genera, such as *Alcaligenes*, *Pseudogracilibacillus*, *Bacillus*, *Proteus*, *Enterococcus*, *Pediococcus*, *Bordetella*, *Pseudomonas*, and *Kerstersia*. With respect to the microbiological risk assessment, attention should be paid to abundant genera, such as *Bacillus*, *Myroides*, *Proteus*, *Providencia*, and *Morganella*, which encompass species described as opportunistic pathogens, bearing drug resistances or causing severe morbidity.

## 1. Introduction

Insects are promising protein sources for livestock and human diet and may represent a sustainable contribution for feeding the growing world population. Larvae and prepupae of *Hermetia illucens* (i.e., black soldier fly, BSF) are receiving increasing interest as potential food and feed source because of the high nutritional value and the low environmental impact of the rearing process [1]. In particular, insect meal of BSF was successfully utilized as an alternative protein source for poultry and aquaculture [2,3,4,5]. The utilization of mature larvae and prepupae in insect-based ingredients (e.g., powders, flours, protein bars, pasta, burgers, and nuggets) requires the development of cheap practices to produce and stabilize the insect biomass, providing a safe starting bulk product [6,7]. The ability of *H. illucens* to grow on a variety of solid organic matrices can be exploited to transform and valorize organic streams such as byproducts of the agroindustry, livestock manures, or urban solid wastes, reducing environmental pollution and converting organic wastes into biomass rich in protein and fat [8,9,10,11].

The possibility to rear *H. illucens* on different waste streams in a biorefinery approach requires attention to safety issues, both chemical and microbiological. The European Food Safety Authority reported the lack of data regarding microbiology, virology, parasitology, and toxicology of insects reared, addressing the potential hazards of nonprocessed insects in comparison with other nonprocessed sources of protein of animal origin [12]. Hygiene issues of edible insects can originate from the substrate but also from the microbial community of the insect gut and may be affected by processing steps linking farming and consumption.

Recent works reported the microbiota composition of BSF larvae and prepupae [3,13,14,15]. However, little attention has been paid to microbial dynamics associated with extrinsic parameters, such as the rearing temperature. From a bioeconomy perspective, the biotransformation of wastes toward new valuable compounds should be a low-energy process that can be exploited in different districts with good tolerance to temperature changes, minimizing the requirement of heating or cooling.

From this standpoint, BSF larvae were reared at different temperatures, to determine the effect of this parameter on the growth rate and the biomass yield of prepupae. Since temperature may be a primary driver of microbial population composition, the microbiota of prepupae reared at 20, 27, and 33 °C was characterized. To avoid biases due to the intrinsic different microbial communities of feed, the larvae were fed a basal substrate [16,17,18]. A survey of the 16S rRNA gene was utilized to determine the microbiota composition in samples of substrate, initial larvae, grown prepupae, and their frass (hereinafter referred to as S, L, PP, and F samples). To assess the risks associated with prepupae utilization as food and feed supplement/ingredient, the metagenomic approach was accompanied by standard microbiology quality control techniques targeting the most relevant food contaminants and pathogens [12,19,20,21]. The impact of rearing temperature on microbiota is expected to offer knowledge and tools to prevent microbiological hazards, protect the health and the welfare of the consumers, and promote the development of safe trade in food and feed products.

## 2. Materials and Methods

### 2.1. Hermetia illucens Rearing

The BSF larvae used in this study were obtained from a colony kept at the Applied Entomology Laboratory, BIOGEST-SITEIA (Reggio Emilia, Italy), originally established with prepupae purchased from CIMI srl (Cuneo, Italy). BSF larvae were routinely reared in a standard vegetable substrate (S), composed of 25% zootechnical use cornflour (mill waste), 15% wheat bran, 10% alfa-alfa flour, and 50% water [22]. The experiments were set up using second to third instar larvae that were obtained from eggs hatched in S and incubated in climatic chambers under controlled conditions of 27 ± 0.5 °C, 65% humidity, and 16:8 light/dark cycles. To compare the rearing temperatures, 100 small larvae (L) were collected and reared in glass containers (20 × 12 × 8 cm) containing 400 g of S within climatic chambers at 20 ± 0.5, 27 ± 0.5, or 33 ± 0.5 °C. Two independent experiments, starting with different batches of L, were carried out, where each rearing temperature was tested in triplicate. Test checks were performed every three days, starting from day seven until the end of the development period, when a minimum of 90% of the initial larvae became prepupae (PP) and the experiment was considered concluded. The time point presenting the highest PP appearance in a single control was registered as the peak of PP. At the end of the experiment, insects were collected from the frass (F). PP and the remaining larvae (L_final_) were counted and weighed to determine total biomass weight, the mean growth rate of each insect, and larvae mortality, according to the following:

(1) $$\mathrm{Total}\;\mathrm{biomass}\;\left(g\right)\;=\;\mathrm{total}\;\mathrm{PP}\;\mathrm{weight}+L_{\mathrm{final}}\;\mathrm{weight}$$

(2) $$\mathrm{Mean}\;\mathrm{growth}\;\mathrm{rate}\;\left(\frac{g}{d}\right)=\frac{\left(\mathrm{mean}\;\mathrm{PP}\;\mathrm{weight}\;+\;\mathrm{mean}\;L_{\mathrm{final}}\;\mathrm{weight}-\;\mathrm{mean}\;L\;\mathrm{weight}\right)}{\mathrm{development}\;\mathrm{period}\;\left(d\right)}$$

(3) $$\mathrm{Larvae}\;\mathrm{mortality}\;\%\;=100\times \left(\mathrm{No}.\;L\;-\mathrm{No}.\;L_{\mathrm{final}}-\mathrm{No}.\;\mathrm{PP}\right)$$

Throughout all the rearing phases, standard hygienic conditions were applied, without using surface disinfection of larvae, substrate sterilization or aseptic incubation. Samples of L, S, PP_20 °C_, PP_27 °C_, PP_33 °C_, F_20 °C_, F_27 °C_, and F_33 °C_ from each experiment were frozen and maintained at –80 °C until analyzed. 

### 2.2. Culture Dependent Microbiological Analyses

Samples consisting of 5 g of S, PP, or F were suspended (10% *w/v*) in buffered peptone water (BD Difco, Franklin Lake, NJ, USA) and homogenized by Ultra Turrax (T25 Ika, Staufen, Germany). Proper dilutions were spread on selective media provided by BD Difco (Franklin Lake, NJ, USA). Total mesophilic aerobes were counted on Plate Count Agar (PCA) after incubation at 30 °C for 72 h. Spore-forming aerobic bacteria were grown on plates of PCA supplemented with 2 g/L of starch incubated at 37 °C for 48 h, with samples heated at 80 °C for 10 min to inactivate vegetative bacteria. Lactic acid bacteria were detected on Lactobacilli MRS agar plates after incubation at 30 °C for 72 h under microaerophilic conditions (GasPack EZ, BD Difco). Enterobacteriaceae were enumerated on Violet Red Bile Agar plates incubated at 37 °C for 24 h. Yeasts and molds were counted on Dichloran Rose Bengal Chloramphenicol agar after incubation at 25 °C for 5 d. Standard methodologies were utilized to detect and enumerate the following food pathogens: *Bacillus cereus*, *Campylobacter* spp.; *Clostridium perfringens*, *Listeria monocytogenes* and *Listeria* spp.; *Salmonella* spp.; and *Staphylococcus aureus,* and coagulase-positive staphylococci [23,24,25,26,27,28,29]. Triplicate samples from the two rearing batches were analyzed (*n* = 6). Microbial contamination was compared with recommendations of international authorities [12,19,20].

### 2.3. 16S rRNA Gene Profiling

For each rearing batch, triplicate samples of L, S, PP_20 °C_, PP_27 °C_, PP_33 °C_, and F_20 °C_, F_27 °C_, and F_33 °C_ were pooled in equal weights. Approximately 2 g of material were 10-fold diluted in PBS and were homogenized by Ultra Turrax. Total DNA was isolated from the suspensions using the DNeasy Mericon Food Kit (Qiagen, Hilden, Germany), following the manufacturer’s standard protocol. The DNA was normalized to 5 ng/µL after quantification with a Qubit v. 3.0 fluorimeter (Thermo Fisher Scientific, Waltham, MA, USA). Partial 16S rRNA gene sequences were amplified using Probio_Uni/Probio_Rev primers, which targeted the V3 region of the 16S rRNA gene. Amplicons were sequenced using a MiSeq (Illumina, San Diego, CA, USA) platform according to Milani et al. [30]. The 16S rRNA gene sequences are available at NCBI repository with the BioProject ID: PRJNA573042.

Raw sequences were cleaned and filtered by size and quality using the software MOTHUR v. 1.25.0 [31]. Quality filtered sequences were processed with QIIME2 pipeline (v. 2019.1) for closed-reference picking of amplicon sequence variants (ASVs), taxonomy assignment, collapsing into operational taxonomic units (OTUs) [32,33]. Closed-reference picking and taxonomy assignation were carried out utilizing as reference SILVA SSU database release 132 (https://www.arb-silva.de/download/arb-files/) with the similarity threshold set at 0.97. The appropriate QIIME2 plugins were utilized to compute the alpha- (observed taxa, Chao1, Shannon, and Pielou’s evenness) and beta diversity (Jaccard, Bray–Curtis, Canberra, Unweighted Normalized UniFrac, and Weighted Normalized UniFrac) and to compare them within and between groups of samples (i.e., the Kruskal–Wallis test for alpha diversity; ANOSIM and PERMANOVA for beta diversity). Beta diversity distance/dissimilarity matrices were utilized for the hierarchical clustering of samples in UPGMA trees and the Principal Coordinate Analysis (PCoA), using QIIME2.

Linear discriminant analysis Effect Size (LEfSe, http://huttenhower.sph.harvard.edu/galaxy) algorithm was applied to discover distinctive taxonomic features characterizing the groups of samples [34]. In the analysis of the taxa characterizing the development stage, L and PP samples were entered as “classes”. In the comparison between frass and insects, F and PP were entered as “classes”. Spearman’s rank correlation was applied to identify the taxa in PP that positively or negatively correlated with the three temperatures. In both LEfSe and Spearman’s analysis, the alpha value for statistical significance was set at 0.05.

### 2.4. Statistics

Plate counts and growth parameters are presented as means ± SD (*n* = 6). ANOVA followed by Tukey’s post-hoc test was carried out with the software SPSS Statistics (v. 21, IBM, Armonk, NY, USA). Differences were considered statistically significant for *p* < 0.05. Spearman’s rank correlation was utilized to estimate the correlation with temperature.

## 3. Results

### 3.1. Growth Performance of Hermetia illucens at Different Temperatures

The growth parameters of *H. illucens* are presented in Table 1. The total development period was the longest at 20 °C (40 d), while it was approximately 24 d at 27 and 33 °C. Rearing at 20 °C resulted in both the lowest total biomass and mean growth rate (15.9 g and 4.1 mg/d). No significant differences were observed between 27 °C and 33 °C (approximately 21 g and 10 mg/d). The highest larval mortality (14.5%) was registered at 33 °C, whereas it was similar at 20 and 27 °C (3.8 and 5.5%, respectively). The temperature significantly affected the time point at which the peak of PP was observed. The higher the temperature, the earlier came the peak (30.0, 19.7, and 16.0 d at 20, 27, and 33 °C, respectively).

### 3.2. Culture Dependent Microbiological Analysis

The viable count of total mesophilic aerobes in PP positively correlated with temperature (ρ = 0.87, *p* < 0.05), being the lowest at 20 °C (7.4 Log_10_ cfu/g) and approximately one magnitude higher at both 27 and 33 °C (Table 2). Aerobic spore-forming bacteria were less abundant than total aerobes, at all the temperatures (*p* < 0.05). Lactic bacteria and Enterobacteriaceae lay in the range of 6.2–6.9 and 3.7–5.2 Log_10_ cfu/g, respectively, without any significant difference associated with temperature. A low load of cultivable yeasts and molds was observed, between 0.7 and 1.2 Log_10_ cfu/g, without significant influence of temperature.

*Bacillus cereus*, *Campylobacter* spp.; *Clostridium perfringens*, coagulase-positive staphylococci, and Listeriaceae were detected in PP reared at all the tested temperatures. The amount of *Campylobacter* and Listeriaceae was positively correlated to temperature (ρ > 0.87, *p* < 0.05). The former passed from 3.2 to 4.7 Log_10_ cfu/g and the latter from 4.8 to 5.8 Log_10_ cfu/g increasing the temperature from 20 to 33 °C. Coagulase-positive staphylococci, *B. cereus*, and *C. perfringens* (laying in the ranges of 3.7–4.2, 2.3–3.2, and 0.8–1.6 Log_10_ cfu/g, respectively) were not affected by the temperature. *Salmonella* spp. was found in 7 out of 18 samples. Among the positive samples, only one (PP grown at 20 °C) allowed the recovery of colonies by direct plating, at a concentration of 1.1 Log_10_ cfu/g.

The microbiological analysis of S revealed a mean viable charge of 4.5 Log_10_ CFU/g total mesophilic aerobes, 4.9 Log_10_ CFU/g aerobic spore-forming bacteria, 2.9 Log_10_ CFU/g lactic acid bacteria, and 3.4 Log_10_ CFU/g fungi, while Enterobacteriaceae, *B. cereus*, *Campylobacter* spp. *C. perfringens*, coagulase-positive staphylococci, and *Salmonella* spp. were absent.

### 3.3. Microbiota Composition by Metagenome Analysis

**Figure 1:**
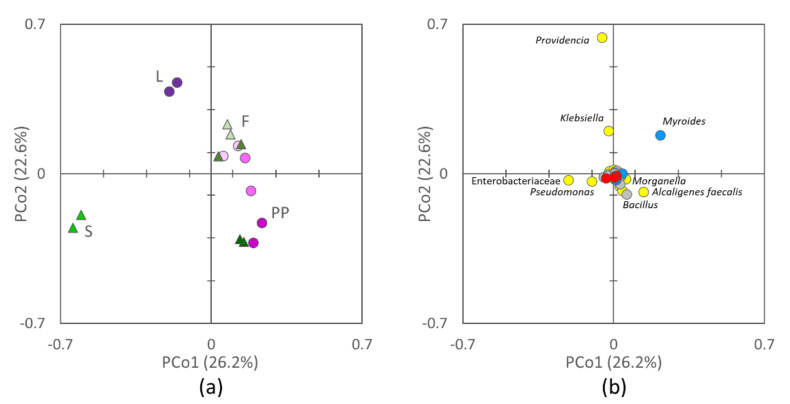
Two-dimensional (2d) Principal Coordinate Analysis (PCoA) visualization of microbiota beta diversity (Bray–Curtis) among initial larvae (L), S, PP, and frass (F): (**a**) scores of L (green circle), S (green triangles), PP (pink circles, with 20, 27, and 33 °C from the lightest to the darkest), and F (pink triangles, with 20, 27, and 33 °C from the lightest to the darkest), (**b**) contribution of the single bacterial taxa (Proteobacteria, yellow; Bacteroidetes, blue, Firmicutes, gray; Actinobacteria, red). Labels indicate taxa with the greatest contribution along PCo1 and PCo2.

The 16S rRNA gene profiling of L, PP, S, and F yielded a total of 755,313 sequences, that were dereplicated into 3974 ASVs hitting a reference sequence in Silva database, and collapsed at the seventh level of taxonomic annotation (i.e., the species, if available) into 536 OTUs. Based on Bray–Curtis distance, the microbiota of L, PP, S, and F clustered in distinct groups (Figure 1) that had different centroids (PERMANOVA, *p* = 0.001) and intragroup permutational similarity significantly greater than the intergroup one (ANOSIM, *p* = 0.001). F represented an exception, the similarity within the group being comparable to that between F and PP (*p* > 0.05). Grouping was evident in the PCo1-PCo2 plot (describing the 49% of total variance), where F and PP were characterized by positive PCo1, L by positive Pco2, and S by negative PCo1. Both F and PP lay at progressively lower PCo2 with the increase of the rearing temperature. Main taxa positively contributing to PCo1 were *Myroides, Alcaligenes faecalis, Bacillus,* and *Morganella*, while Enterobacteriaceae weighted negatively. Positive PCo2 was strongly owed to *Providencia*, followed by *Klebsiella* and *Myroides*, and negative to *Bacillus, Pseudomonas*, and *A. faecalis*. 

The richness of the microbiota (evaluated as total no. of OTUs and Chao1 index) was similar in all the groups of samples, regardless of the rearing temperature (Supplementary Figure S1). The evenness (estimated in Shannon and Pielou metrics) was the highest in S samples, followed by PP and F, grouped regardless of the rearing temperature, and the lowest in L (Supplementary Figure S1).

**Figure 2:**
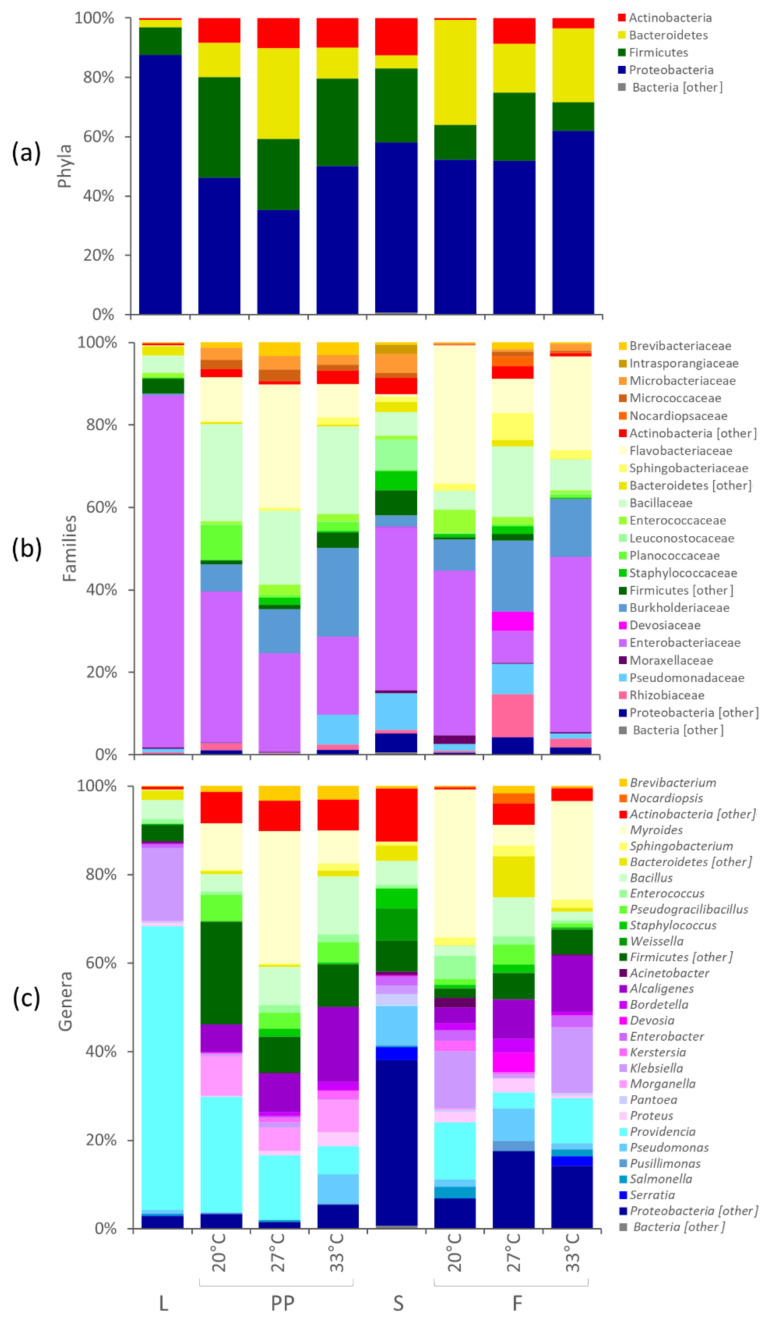
Stacked bar-plot representation of microbiota composition in L, S, PP, and F with taxonomic features collapsed at the level of phyla (**a**), families (**b**), and genera (**c**). The mean values of the two independent experiments are reported. The phyla, families, and genera that remained unclassified or never occurred with abundance >2.0% are grouped as others.

**Figure 3:**
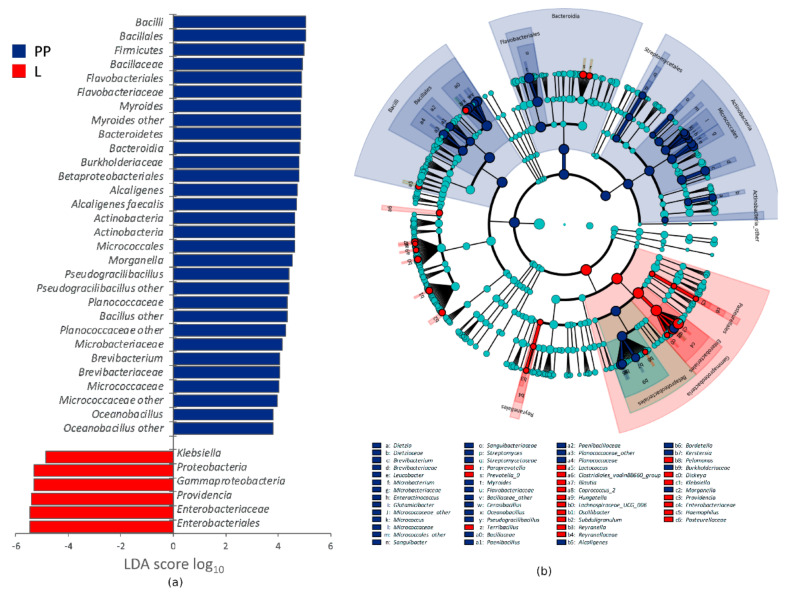
Linear discriminant analysis Effect Size (LEfSe) analysis of taxonomic features differentiating L (*n* = 2) and PP (*n* = 6), regardless of the growth temperature: (**a**) LDA logarithmic scores of taxonomic biomarkers exhibiting significant differential abundance (*p* < 0.05, logarithmic LDA logarithmic score ≥ 2.0) and appearing at least once with abundance >2.0%; (**b**) cladogram of all the taxonomic biomarkers.

A reduced dataset of 181 OTUs, occurring for more than 0.2% in at least one sample, still represented 99% of the reads. The distribution of the OTUs in the groups of samples is reported in the Venn diagram of Supplementary Figure S2. The bacterial composition of L, PP, S, and F is reported in Figure 2, showing the mean abundance of the main bacterial groups in the two experiments. The two experiments were not averaged for the analysis of the differential abundance of bacterial groups and the effect of temperature. The microbiota of L encompassed 98 of the 181 OTUs. It was dominated by Proteobacteria (87.4%), with remarkably high amounts of *Providencia* (64.1%) and *Klebsiella* (16.4%), followed by Firmicutes (9.3%), especially *Bacillus* (4.3%). The microbiota of PP included 156 OTUs, 95 of which shared with L. PP were poorer than L in Proteobacteria and richer in Bacteroidia, Actinobacteria, and Bacilli (Figure 2 and Figure 3). *Providencia* remained abundant in PP (from 6.4 to 26.1%, depending on the temperature), although it significantly decreased compared to L, likewise *Klebsiella* and other minor Enterobacteriaceae. The genera *Morganella*, *Alcaligenes*, *Bordetella*, and *Kerstersia* behaved in contrast to most Proteobacteria and were significantly enriched in PP. *Morganella* and *Alcaligenes*, in particular, reached remarkable levels (8.9 and 16.9%, respectively). The significant increase of Bacteroidia in PP was mainly due to *Myroides*, a Flavobacteriaceae that reached 30%. *Bacilli* also included several biomarkers characterizing PP, such as Planococcaceae, Paenibacillaceae, *Pseudogracilibacillus*, *Oceanobacillus*, and unclassified members of the genus *Bacillus*. Planococcaceae, *Pseudogracilibacillus,* and unclassified *Bacillus* reached 8.5, 5.9, and 13.1%, respectively. Within Actinobacteria, the main biomarkers characterizing PP were Micrococcales belonging to *Brevibacterium*, which increased up to 3.3%, and several minor genera.

The microbiota of S was characterized by 133 OTUs, 85 of which shared with L and 109 shared with PP. The remaining OTUs represented 6.3% of S microbiota, with only *Rahnella* being >1%. The microbiota of F encompassed 142 OTUs, 139 of which shared with PP. The composition of F differed from that of PP for being significantly richer in Proteobacteria (such as *Klebsiella* and *Acinetobacter*) and poorer in Actinobacteria (mainly Micrococcales, such as *Brevibacterium*), Firmicutes (Bacillales) and the Enterobacteriaceae *Morganella* (Supplementary Figure S3).

**Figure 4:**
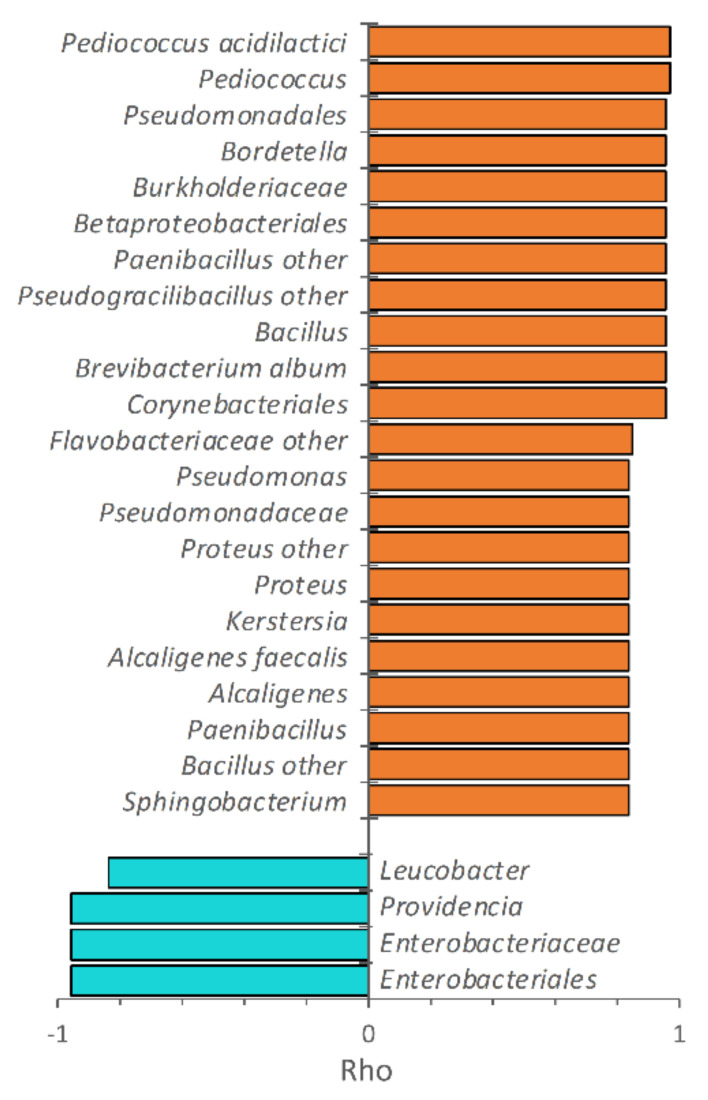
Spearman’s rank correlation of the bacterial taxa in PP and the growth temperature. The correlation coefficient rho is reported only for the taxa exhibiting a positive (orange) or negative (cyan) significant correlation with temperature and appearing at least once with abundance >1.0% (*n* = 2, *p* < 0.05).

The increase of incubation temperature negatively correlated with the level of Enterobacteriaceae in PP, mostly due to the decrease of *Providencia* (Figure 4). Unlike *Providencia*, other Proteobacteria positively correlated with temperature, such as *Kertersia, Proteus*, *Pseudomonas*, *Alcaligenes,* and *Bordetella*. *Pseudomonas* was abundant only in PP reared at 33 °C (6.7%), whereas at the lower temperatures it accounted for less than 0.1%. *Bacillus* was also positively associated with increasing temperature and reached 13.1% at 33 °C. Increasing temperatures also favored the populations of other Actinobacteria (*Brevibacterium*), Bacilli (e.g., *Enterococcus*, *Pediococcus*, *Paenibacillus*, *Pseudogracilibacillus*), and Bacteroidetes (*Sphingobacterium*), although occurring in low amounts. The correlation of taxa with temperature is in agreement with their contribution in the PCoA biplot, resulting in PP samples being located at lower PCo1 values with the increase of the rearing temperature (Figure 1).

## 4. Discussion

In the present study, the larvae of *H. illucens* were reared at three different temperatures (20, 27, and 33 °C), utilizing a standard vegetal substrate, until reaching the PP stadium. The larvae efficiently grew across a range of temperatures, although with differences in the development rate, confirming the robustness of *H. illucens*. In the tropics, the development of *H. illucens* occurs yearlong, while it is restricted to a few generations per year in warm temperate regions [35], generally requiring temperatures above 26 °C [36]. Higher temperatures promoted faster larval development and greater final biomass but also higher mortality, in agreement with previous studies [37,38,39], suggesting that the best rearing temperature is 27 °C. The flexible rearing temperature enables the use of these insects also in developing countries, with minimal investment in energy-consuming devices for cooling or heating.

The first step of microbiological risk assessment in food or feed involves the collection of all information pertaining to potential pathogens that may exert any adverse effects on human or animal health. In the present study, the effect of rearing larvae at different temperatures on the microbiota of *H. illucens* was evaluated, with a focus on the viable load of the main food pathogens according to the Advisory Reports 2014/2372 and 2019/6200 [19,20]. The rearing temperature slightly affected the microbial load of PP, with only total mesophilic aerobes and Enterobacteriaceae significantly lower at 20 °C than at the higher temperatures. All the searched pathogens were detected in the whole set of PP samples and the counts of *Campylobacter* and Listariaceae got higher with the increasing temperature. Interestingly, the substrate was negative to all the pathogens except Listeriaceae. The larvae were not disinfected and were not reared under aseptic conditions, according to hygienic conditions that may be achieved in a production facility, where the substrate (e.g., a nonsterilized vegetal waste) is expected to be the main source of microbial contamination. It is likely that Enterobacteriaceae, *B. cereus*, *Campylobacter* spp., *C. perfringens*, coagulase-positive staphylococci, and *Salmonella* spp., laying below the limit of detection in S, were environmental contaminants or were introduced with small larvae. In any case, hygienic conditions must be kept under control throughout the multiple manufacturing processing stages, because the rearing of PP favored the blooming of the pathogens. Major attention has to be paid to microbiological optimization through the processes aimed at stabilizing the insects, such as powdering, heating, drying, UV treating, high-energy microwaving, pasteurizing, and acidifying, which are expected to contain the load of pathogens.

Metagenome analysis was complementary to the outcome of traditional microbiological approaches, providing more comprehensive information on ecological aspects. Previous studies determined the microbiota composition of *H. illucens* by 16S rRNA gene profiling, focusing on entire specimens, with or without any surface sterilization, or on the dissected gut, in some cases distinguishing specific gut sections [13,15,16,17,18,40]. These studies pointed toward a great variability of *H. illucens* microbiota, with the substrate, the stage of development of the insect, and the gut section being the major players shaping the structure and the diversity of the bacterial community. In some cases, the microbiota of *H. illucens* was dominated by Bacteroidetes, in others by Proteobacteria, accompanied by very variable amounts of Actinobacteria and Firmicutes [13]. However, the existence of a core composition, independent of the substrate and transmitted through the development stages, seems plausible. In particular, *Providencia* was reported as one of the most recurrent and abundant genera, occurring in insects reared on different substrates. Consistently, in the present study, the microbiota of L was dominated by *Providencia* (>60%) and other Proteobateria (mainly *Klebsiella*) and evolved to a more complex composition with insect development. A lower amount of *Providencia* was still present in the microbiota of PP, while Actinobacteria, Bacteroidetes, and Bacilli bloomed (Figure 2). Many taxa dominating the microbiota of PP (e.g., *Providencia*, *Alcaligenes faecalis*, *Myroides*, *Morganella*, *Bordetella*, and *Kerstersia*) occurred also in L and were not found or were negligible in S. These taxa took the most advantage from the interaction with the insect, exhibiting the greatest fitness relative to *H. illucens*. Other taxa, such as *Pseudogracilibacillus* and other unclassified Bacillaceae, that colonized PP were not found in either L or S. It is not clear whether they were contaminants of the rearing environment or they initially lay below the limit of detection in L and S.

For the first time, a significant effect of the rearing temperature on the microbiota composition of *H. illucens* is reported. The temperature was negatively associated with the amount of *Providencia*, which was the lowest at 33 °C. On the contrary, the temperature was positively associated with a variety of other genera, such as *Alcaligenes*, *Pseudogracilibacillus, Bacillus, Proteus, Enterococcus*, *Pediococcus*, *Bordetella, Pseudomonas*, and *Kerstersia*. Thus, the temperature has to be included among the main factors affecting the microbiota and may have contributed, together with the rearing substrate, to the wide differences in the structure of the microbial community associated with *H. illucens* reported in the literature.

The present study also indicates that *H. illucens* largely shares the microbiota with the medium where it is reared, except for a few lowly represented taxa. The main taxa constituting the microbiota of F and PP were the same, although some of them presented a differential abundance between the two environments, confirming that the insects and the medium shape the microbiota of each other [16].

With respect to the microbiological risk assessment, attention should be paid to the relevant abundance of some genera, such as *Myroides*, *Proteus*, *Providencia*, and *Morganella*, revealed by the 16S rRNA gene profiling. Species within these genera are not considered primary pathogens, although there is a vast literature describing them as opportunistic pathogens that may bear drug resistances and may cause severe morbidity [41,42,43,44,45]. Characterizing the species of *Myroides*, *Proteus*, *Providencia*, and *Morganella* harbored by *H. illucens* is recommended to establish whether they could be capable of causing adverse effects if present in the final product or throughout the production process.

## Figures and Tables

**Table 1:** Growth parameters of *H. illucens* at the three temperatures tested (definitions in materials and methods). Within each line, values marked with different letters indicate significant differences (ANOVA followed by Tukey’s post-hoc test, *p* < 0.05).

Growth Parameter	20 °C	27 °C	33 °C
Development period (d)	40.0 ± 0 ^a^	24 ± 1.1 ^b^	23.7 ± 1.0 ^b^
Total biomass (g)	15.9 ± 2.3 ^b^	21.3 ± 3.8 ^a^	21.0 ± 1.3 ^a^
Mean growth rate (mg/d)	4.1 ± 0.6 ^b^	9.4 ± 1.5 ^a^	10.4 ± 0.7 ^a^
Larvae mortality (%)	3.8 ± 2.0 ^b^	5.5 ± 5.3 ^b^	14.5 ± 7.4 ^a^
Peak of PP (d)	30.0 ± 1.5 ^a^	19.7 ± 2.6 ^b^	16.0 ± 1.6 ^c^

**Table 2:** Cultivable microorganisms observed in substrate (S) and grown prepupae (PP) grown at 20, 27 and 33 °C. Counts are expressed as Log_10_ CFU/g. For *Salmonella* spp. the number of positive samples out of six is reported.

Viable Counts	S		PP		Contamination Limits ^1^
		20 °C	27 °C	33 °C	
Total mesophilic aerobes	4.5 ± 0.2	7.4 ± 0.9 ^b^	8.5 ± 0.4 ^a^	8.7 ± 0.3 ^a^	<5.7
Aerobic spore-forming bacteria	4.9 ± 0.8	6.8 ± 1.3 ^a^	7.9 ± 0.5 ^a^	7.9 ± 0.2 ^a^	n.s.
Lactic acid bacteria	2.9 ± 0.7	6.6 ± 0.4 ^a^	6.9 ± 0.7 ^a^	6.2 ± 0.5 ^a^	n.s.
*Enterobacteriaceae*	-	3.7 ± 0.3 ^b^	5.2 ± 0.6 ^a^	4.8 ± 1.3 ^ab^	<3.0
Yeasts and molds	3.4 ± 0.3	0.7 ± 0.9 ^a^	1.2 ± 1.0 ^a^	0.9 ± 0.8 ^a^	n.s.
*Bacillus cereus*	-	2.3 ± 0.9 ^a^	3.2 ± 0.7 ^a^	2.3 ± 0.7 ^a^	<5.0
*Campylobacter* spp.	-	3.2 ± 0.3 ^c^	4.2 ± 0.4 ^b^	4.7 ± 0.2 ^a^	absent in 25 g
*Clostridium perfringens*	-	0.8 ± 0.7 ^a^	1.6 ± 1.3 ^a^	1.0 ± 0.8 ^a^	<5.0
Coagulase-positive staphylococci	-	3.9 ± 0.7 ^a^	4.2 ± 0.7 ^a^	3.7 ± 0.9 ^a^	<5.0
*Listeriaceae*	2.6 ± 0.4	4.8 ± 0.4 ^b^	5.5 ± 0.3 ^a^	5.8 ± 0.4 ^a^	<2.0
*Salmonella* spp.	0/6	2/6	1/6	4/6	absent in 25 g

Values are means ± SD (*n* = 6). - indicates < 2 Log_10_ CFU/g in the 6 replicates. For PP, within each line, values marked with different letters indicate significant differences (ANOVA followed by Tukey’s post-hoc test, *p* < 0.05). ^1^ Reference values according to NVVWA and EFSA [12,19,20]. n.s., limit not specified.

## Supplementary Materials

The following are available online at https://www.mdpi.com/2076-2607/8/6/902/s1, Figure S1: Alpha diversity metrics of the microbiota in L, PP, S, and F; Figure S2: Distribution in the main 181 OTUs in L, PP, S, and F samples; Figure S3: LEfSe analysis of taxonomic features differentiating F and PP.
