# Supplementary material for: Effect of Rearing Temperature on Growth and Microbiota Composition of Hermetia illucens
Source: Microorganisms. 2020 Jun 15;8(6):902. doi: 10.3390/microorganisms8060902 (PMC7355568; doi:10.3390/microorganisms8060902)
Supplement: Supplementary file 1 [file microorganisms-08-00902-s001.pdf]

microorganisms

MDPI

Article

Effect of rearing temperature on growth and microbiota composition of *Hermetia illucens*

Stefano Raimondi [1], Gloria Spampinato [1], Laura Ioana Macavei [1], Linda Lugli [1], Francesco Candeliere [1], Maddalena Rossi [1,2], Lara Maistrello [1,2], Alberto Amaretti [1,2,*]

1 Department of Life Sciences, University of Modena and Reggio Emilia, 41125 Modena, Italy
2 BIOGEST – SITEIA, University of Modena and Reggio Emilia, 42124 Reggio Emilia, Italy
* Correspondence: alberto.amaretti@unimore.it; Tel.: +39-059-205-8588

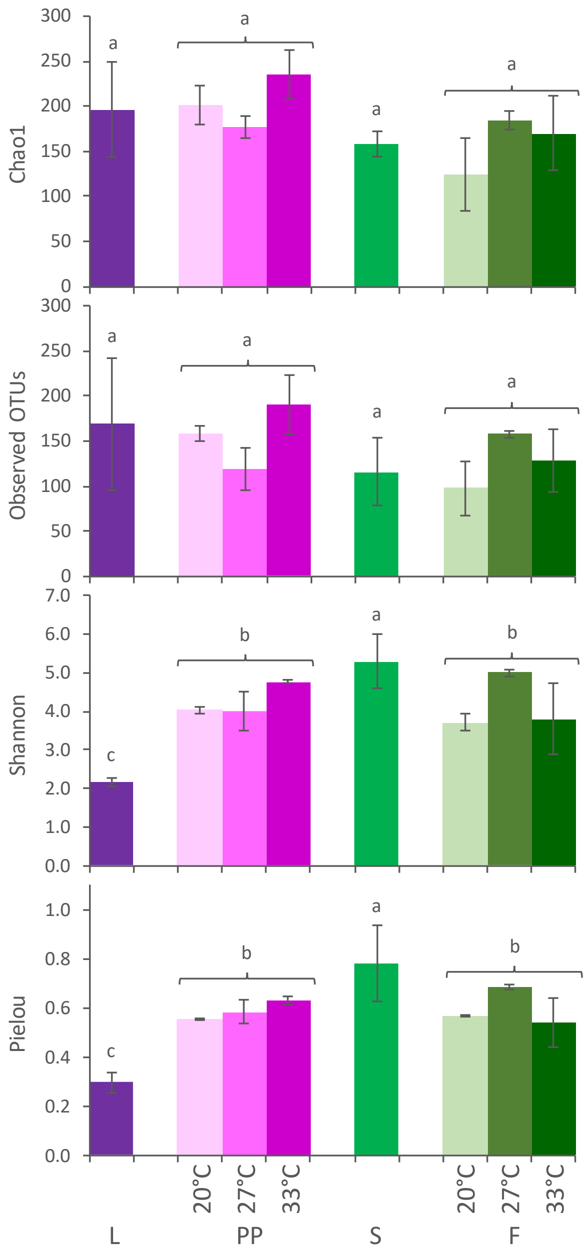

Supplementary Figure S1. Alpha diversity metrics of the microbiota in L, PP, S, and F. Groups significance: within each panel, values sharing the same letter do not significantly differ (Kruskal-Wallis, P > 0.05); PP and F were grouped regardless of the rearing temperature.

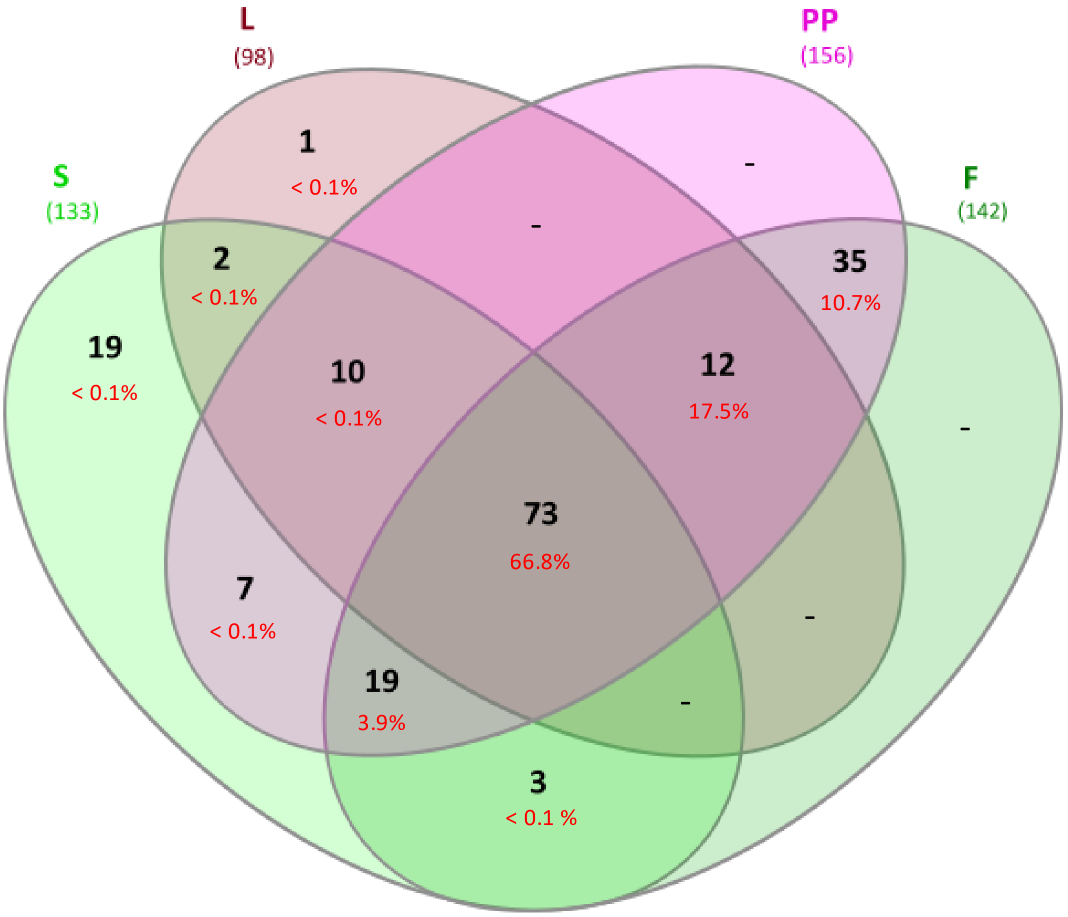

Supplementary Figure S2. Distribution in the main 181 OTUs (accounting for more than 0.2% in at least one sample) in L, PP, S, and F samples. For each intersection, the number of OTUs and the percentage relative to the total reads are reported in black and red, respectively.

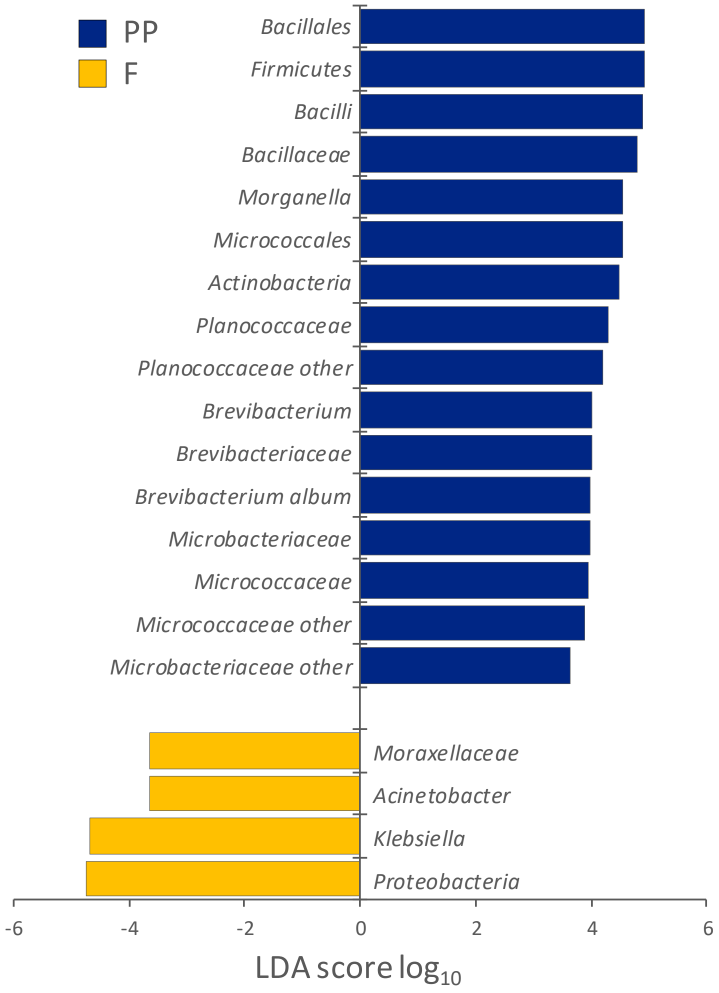

Supplementary Figure S3. LEfSe analysis of taxonomic features differentiating F and PP (both n = 6), regardless of the growth temperature. The plot reports LDA logarithmic scores of taxonomic biomarkers exhibiting significant differential abundance ($P < 0.05$, logarithmic LDA logarithmic score ≥ 2.0) and appearing at least once with abundance > 2.0%.
